# The RNA world of fungal pathogens

**DOI:** 10.1371/journal.ppat.1011762

**Published:** 2023-11-30

**Authors:** Srimeenakshi Sankaranarayanan, Seomun Kwon, Kai Heimel, Michael Feldbrügge

**Affiliations:** 1 Heinrich-Heine University Düsseldorf, Institute for Microbiology, Cluster of Excellence on Plant Sciences, Düsseldorf, Germany; 2 Georg-August University Göttingen, Institute for Microbiology and Genetics, Göttingen Center for Molecular Biosciences (GZMB), Göttingen, Germany; University of Maryland, Baltimore, UNITED STATES

Fungal pathogens execute well-defined infection programs that are intensively regulated at the posttranscriptional level. RNA biology determines the precise timing and subcellular expression of the encoded proteins. This temporal coordination is especially vital for membrane trafficking in order to synchronize the intracellular organelle network. This includes vacuole maturation, intra- and extracellular vesicle (EV) transport, protein entry into mitochondria, peroxisomes, and the ER. Here, we explore the intimate link between membrane trafficking, organelle function, and the RNA world during fungal plant pathogenesis, with a specific emphasis on the corn smut *Ustilago maydis*.

## Endosomal mRNA transport generates and prevents intracellular gradients in infectious hyphae

**Fig 1:**
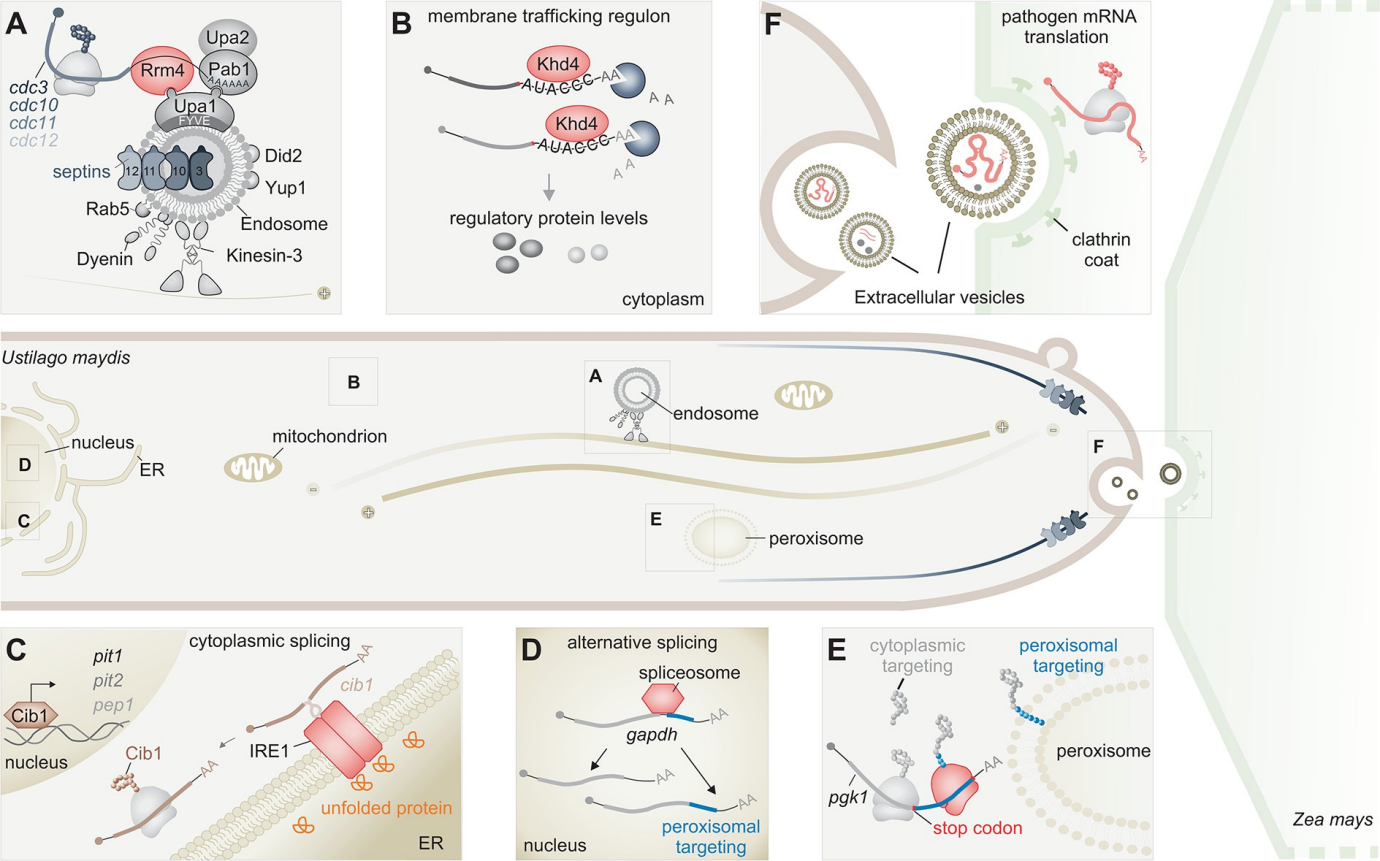
In fungal hyphae, mRNA-dependent processes drive membrane trafficking. Schematic depiction of *U*. *maydis* hyphae (left) interacting with its plant host, *Zea mays* (right). Distinct cellular processes within the hyphae have been marked and magnified. (**A**) Early endosomes act as a platform for the transport of mRNAs. Rrm4 (red) facilitates the mRNA transport by hitchhiking onto Rab5-positive early endosomes. Interactions between Rrm4, the poly (A)-binding protein, Pab1, and the FYVE domain-containing adapter protein Upa1 enable the attachment of mRNAs to early endosomes. The scaffold protein Upa2 stabilizes the transport complex. This bidirectional movement relies on motor proteins, kinesin, and dynein, traveling along antiparallel microtubules. The local translation of the cargo mRNAs like *septin* (blueish gray) on early endosomes is essential to generate higher-order septin filaments extending from the hyphal tip. (**B**) Khd4 (red) orchestrates membrane trafficking by regulating the subcellular levels of regulatory proteins crucial for this process. The RBP coordinates the destabilization of mRNAs encoding membrane trafficking regulators (gray) into an mRNA regulon through the recognition of the AUACCC motif present in their 3′ UTRs. This mRNA decay process determines the induction kinetics of these mRNAs, fine-tuning their steady-state levels following transcriptional induction. Pacmans in bluish-gray depict mRNA decay factors. (**C**) Unconventional splicing of *cib1* mRNA in the cytoplasm elicits the UPR, required to preserve ER homeostasis during infection. Upon ER stress, the ER membrane-localized Ire1 kinase/RNAse (red) is activated through its direct interaction with unfolded or misfolded proteins (orange) or by reduced Ire1-Bip1 interaction. This activation prompts the splicing of an intron from the *cib1* mRNA (brown) in the cytoplasm. The resulting spliced *cib1* mRNA is translationally active, leading to the production of the Cib1 protein (brown)—a bZIP transcription factor, which in turn, induces the expression of UPR genes to restore ER homeostasis and secretion. These include genes such as *pit1*, *pit2*, and *pep1*, encoding effector proteins vital for fungal infection. (**D**, **E**) Posttranscriptional regulation determines the dual targeting of peroxisomal proteins. (**D**) Alternative splicing of *gapdh* mRNA leads to the expression of an isoform containing peroxisomal targeting sequence (blue), which facilitates peroxisomal targeting after translation. The spliceosome complex is represented in red (*gapdh-*glyceraldehyde-3-phosphate dehydrogenase). (**E**) Ribosomal readthrough past stop codon (red box) of *pgk1* mRNA integrates cryptic peroxisomal targeting sequence (blue) at the C-terminus, facilitating peroxisomal entry. The ribosome executing the stop-codon readthrough is depicted in red (*pgk1*-3-phosphoglycerate kinase). (**F**) Fungal mRNAs (red) encoding effector proteins are packaged within EVs as a means to transport into the host plant. The internalization of these fungal EVs into the plant host (light green) might be aided by clathrin-mediated endocytosis. Following their entry into plant cells, the delivered fungal mRNAs are subsequently translated using the plant’s own translation machinery, thus outsourcing the production of fungal effector proteins within the plant for pathogenic development. In addition to RNAs, the cargoes of fungal EVs encompass proteins, and secondary metabolites, both depicted in gray.

Many fungal pathogens require regulation of hyphal morphology for host invasion. These highly polarized hyphae rely on active transport along the cytoskeleton to deliver proteins to distinct subcellular regions. A fundamental mechanism is the transport of mRNAs, providing precise spatial and temporal control of protein synthesis. RNA-binding proteins (RBPs) play central roles in dictating the fate of mRNA from synthesis to degradation. Key RNA-binding proteins, for example, She3 and Rrm4, mediate actin- and microtubule-dependent mRNA transport in *Candida albicans* and *U*. *maydis*, respectively [1,2]. Importantly, studying Rrm4 has uncovered a novel transportation mechanism: mRNAs hitchhike on the cytoplasmic surface of endosomes, linking mRNA and membrane trafficking [2]. Rrm4 harbors 3 N-terminal RRM domains for RNA-binding, with notable cargo mRNAs encoding all 4 *septins*: Cdc3, Cdc10, Cdc11, and Cdc12 [3]. During transport, endosome-coupled translation ensures the formation and assembly of heterooctameric septin complexes on endosomes. These complexes are ultimately delivered to the growth pole, resulting in the formation of septin filaments with a defined gradient emanating from the hyphal tip (Fig 1A).

A transcriptome-wide iCLIP analysis revealed that Rrm4 interacts with hundreds of mRNAs, primarily through their 3′ UTR [3]. This finding highlights the critical role of endosomal mRNA transport in efficiently distributing bulk mRNAs and associated ribosomes throughout hyphae, thereby preventing concentration gradients around the nucleus [4,5]. Interestingly, a substantial number of mRNAs encoding mitochondrial proteins contain the Rrm4 binding site within their 3′ UTR. Thus, Rrm4-mediated mRNA transport and endosome-coupled translation might facilitate mitochondrial protein entry, similar to processes observed in polar growing neurons [6].

An outstanding question is how mRNAs are linked to transport endosomes. In plants, endosomal transport RBPs directly interact with the small GTPase Rab5, while in animal cells, the FERRY complex and annexin have been implicated in this process [5]. In *U*. *maydis*, the scaffold protein Upa2 interacts with the poly(A)-binding protein, Pab1, to stabilize endosomal mRNA transport complexes [7]. Furthermore, the C-terminus of Rrm4 contains a novel binding platform comprising 3 MLLE domains that operate in a strict hierarchy: while MLLE1 and MLLE2 serve accessory functions, MLLE3 is essential for the endosomal attachment of Rrm4 [8]. Specifically, MLLE3 recognizes 2 PAM2-like sequences in the endosomal adaptor protein Upa1, which, through its FYVE zinc finger domain, anchors the mRNP complex to the endosome by recognizing phosphatidylinositol 3-phosphate, the characteristic lipid of early endosomes (Fig 1A). Recently, the first evidence of endosomal mRNA transport was also discovered in hyphae of *Sordaria macrospora* [5] suggesting that related fungal pathogens, such as *Aspergillus fumigatus*, may also utilize this mRNA transport process for hyphal development.

## Regulation of mRNA stability determines membrane trafficking dynamics

Human pathogens such as *Cryptococcus neoformans* and *C*. *albicans* as well as plant pathogens such as *U*. *maydis* regulate RBP-mediated mRNA stability to control their virulence [9,10]. In *U*. *maydis*, the multi-KH domain RBP Khd4 is crucial for morphology and pathogenicity [11]. A transcriptome-wide target mRNA detection analysis was performed using hyperTRIBE (Targets of RBP Identification By Editing). In this method, Khd4 was fused with the hyperactive version of the heterologous RNA-editing enzyme ADAR (Adenosine Deaminase Acting on RNA), enabling the marking of target mRNAs through adenosine editing, which were subsequently identified using high-throughput sequencing. This in vivo approach, revealed that Khd4 binds mRNAs encoding regulatory proteins involved in membrane trafficking and specifically interacts with AUACCC regulatory elements in their 3′ untranslated region (UTR) triggering degradation [10]. Without this regulation, target mRNAs are stabilized, leading to a delayed response time during hyphal induction and pathological overshooting of their expression levels (Fig 1B). As a consequence, membrane trafficking dynamics are disrupted, causing aberrant vacuole biogenesis, and temporally altered hyphal polar growth. Hence, Khd4 unites target mRNAs encoding regulatory proteins into a defined membrane trafficking RNA regulon to ensure coordinated control of intracellular trafficking during hyphal growth [10]. Given the significance of proper vacuole biogenesis in the development of appressoria-like infection structures in phytopathogens, the regulatory potential of Khd4 expands the influence of RBPs in affecting pathogen entry into plant hosts.

## Cytoplasmic mRNA splicing coordinates UPR and ER trafficking during infection

Another tight link between RNA biology, protein, and membrane trafficking is the regulation of the unfolded protein response (UPR) mediating ER homeostasis during secretion. Unconventional splicing of *cib1* mRNA occurs on the cytoplasmic surface of ER membranes and is mediated by the transmembrane kinase/RNAse Ire1 in response to ER stress (Fig 1C). The mature mRNA encodes the key bZIP transcription factor eliciting UPR. Importantly, human- and plant-pathogenic fungi rely on a functional UPR for virulence, as it plays a central role in thermotolerance, antifungal drug resistance, and proper protein secretion [12]. In *U*. *maydis*, the UPR is adapted to the biotrophic lifestyle of the fungus and exerts additional virulence-related functions that connect cellular physiology with the development and even transcriptional regulation of host-manipulating effectors.

During the pathogenic program, the UPR is specifically activated after plant invasion. This correlates with increased expression of more than 200 effectors many of which follow the classical ER-dependent secretory pathway [13]. During infection, the interaction between Cib1 and the regulatory protein Clp1 promotes mutual stabilization and accumulation of both proteins [14,15]. As Clp1 levels are decisive for initiating fungal proliferation [16,17], this interaction ensures elevated secretory capacities of fungal cells prior to host colonization. In parallel, the Cib1/Clp1 complex promotes modulated UPR gene expression, enabling long-term UPR activity [14]. Transcriptome-wide analysis revealed comprehensive up-regulation of genes involved in membrane transport and protein processing in the ER [15], consistent with the increased demand for membrane traffic and effector delivery throughout infection.

Excessive ER stress is suppressed by the negative feedback of the UPR on the signaling network, which controls the expression of the vast majority of effector genes [13]. Importantly, some effector genes are a direct target of the UPR regulator Cib1 [18] and thus may escape this global repression, arguing for an important contribution of these effectors in virulence. Indeed, 3 of the 4 directly regulated effector-related genes encode essential virulence factors (Fig 1C; *pit1*, *pit2*, *pep1*) [13]. Interestingly, another mode of fine-tuning UPR kinetics is found in *C*. *neoformans*. Here, binding of the pumilio-type RNA-binding Puf4 to the mRNA encoding the Cib1-like protein Hxl1 supports unconventional splicing during UPR activation and mRNA degradation during recovery from ER stress [19]. In essence, RNA regulation at the level of unconventional splicing is upstream of a defined transcriptional response regulating correct ER-dependent membrane trafficking.

## Diverse RNA regulation determines peroxisomal protein entry

Peroxisomes are essential organelles involved in myriad cellular processes including lipid homeostasis, reactive oxygen metabolism, and secondary metabolite production. In *U*. *maydis*, several glycolytic enzymes such as glyceraldehyde-3-phosphate dehydrogenase (GAPDH) and 3-phosphoglycerate kinase (PGK-1) are targeted to peroxisomes through alternative splicing and translational readthrough, respectively. In both cases, the C-terminus gains a functional peroxisomal targeting sequence (Fig 1D and 1E) [20]. As peroxisomal membranes are impervious to NAD^+^/NADH, the cryptic peroxisomal localization of these enzymes coordinates redox cycling between cytoplasm and peroxisomes. Significantly, stop-codon readthrough stands out as the predominant mechanism employed by both fungi and mammals to generate cryptic peroxisomal targeting sequences, whereby ribosomes continue translating past a termination codon to produce peroxisomal isoforms. This low rate of stop-codon readthrough rates enables the release of small but steady amounts of these enzymes to support peroxisome metabolism [21,22]. For translational readthrough, both *U*. *maydis* and human cells require a specific sequence context involving the UGA stop codon followed by the dinucleotide CU [23]. A recent discovery revealed another mechanism involving translation initiation with non-AUG start codons that target 6-phosphogluconate dehydrogenases of the pentose phosphate pathway to peroxisome [24]. Notably, removing the peroxisomal targeting sequence from these bona fide cytosolic enzymes reduces the virulence of *U*. *maydis*, underscoring the significance of RNA regulation-dependent peroxisomal protein targeting in pathogenic development.

## Extracellular vesicle-mediated transport of RNAs determines host communication

Intracellular membrane and RNA trafficking processes can influence the extracellular milieu via secretion. Pathogenic fungi secrete molecular weapons, termed effectors, to manipulate the host during infection. Extracellular RNAs (exRNAs) are emerging as a novel class of effectors that can modulate host gene expression. The phenomenon of bidirectional cross-kingdom RNAi (ckRNAi) has been particularly well documented in plant–pathogen interactions, whereby both partners secrete small RNAs (sRNAs) that silence genes in one another [25]. The first sRNA effectors were discovered in *Botrytis cinerea*, which is loaded into AGO1-RISC of the host *Arabidopsis thaliana* to down-regulate plant defense-related genes [26]. ckRNAi is a widespread phenomenon; sRNA effectors have been found in various other fungal, oomycetes, and even in bacterial pathogens and symbionts [25].

The routes of secretion and uptake of RNA during host–pathogen interaction are contentious, due to several possible mechanisms and a large variability between the systems. Extracellular vesicles (EVs) are one of the ubiquitous means of RNA secretion. In plant–pathogen interactions, *A*. *thaliana* sRNAs targeting *B*. *cinerea* transcripts were found associated with EVs, and DEAD-box RNA helicases and annexins have been implicated in their secretion [27]. As for the uptake of exRNAs, clathrin-mediated endocytosis of fungal EVs is a likely route of fungal sRNA effector delivery, but it remains to be elucidated how these sRNAs escape endosomes and are loaded into host AGOs [25].

Besides canonical sRNAs and mRNAs, tRNAs and tRNA fragments, circular RNAs, and even rRNAs can be secreted [25]. As EVs can carry much larger RNAs, there is an intriguing possibility of fungal mRNA delivery and translation into functional effector proteins in host cells (Fig 1F). EV-associated mRNAs of *Paracoccidiodes brasiliensis* are translation-competent, yielding protein products in vitro. A comprehensive analysis of mRNAs associated with EVs from *U*. *maydis* cultures, designed to mimic infectious hyphae, has identified several candidate mRNA effectors that meet the following criteria: relatively enriched in EVs compared to the cells and up-regulated during infection [28]. As expected for a pathway parallel to conventionally secreted effectors, ER-targeted mRNAs and those encoding proteins with signal peptides were underrepresented in EVs. Notable among mRNA effector candidates are those encoding metabolic enzymes, which may reflect the capacity of *U*. *maydis* to reprogram the host metabolism [28]. The biological relevance of these diverse RNA species from pathogens remains to be discovered, whether they serve as virulence factors or elicitors of host immunity during interaction with the host, or alternatively as mediators of intraspecies communication between fungal cells.

## Conclusions and future directions

Studying *U*. *maydis* as a model pathogen reveals an intricate interplay between RNA biology and membrane trafficking. The regulation of RNA processes like splicing, localization, translation, and stability, precisely governs the exact translation levels of virulence factors. This investigation unveils a novel connection involving small GTPase regulation, septins, UPR, peroxisomal function, and the pathogen RNA trafficking to host cells.

Understanding RNA biology is essential for studying the fundamental biology of fungal pathogens. A recent demonstration showcased that in *Magnoporthe oryzae* tRNA modification and codon usage regulate unconventional secretion of protein effectors [29]. Advances in techniques like iCLIP and HyperTRIBE have propelled the investigation of RBPs in fungal pathogens, enabling detailed mapping of RBP-binding across the transcriptome. HyperTRIBE, in particular, offers a unique avenue to delve into the host–pathogen interaction interface at the RNA level. These methods promise discoveries that advance fungal pathogenesis understanding, fostering novel antifungal therapies.

## References

[1] Elson SL, Noble SM, Solis NV, Filler SG, Johnson AD. An RNA transport system in *Candida albicans* regulates hyphal morphology and invasive growth. PLoS Genet. 2009;5(9):e1000664. doi: 10.1371/journal.pgen.1000664 . 19779551 PMC2739428

[2] Béthune J, Jansen RP, Feldbrügge M, Zarnack K. Membrane-associated RNA-binding proteins orchestrate organelle-coupled translation Trends in cell biology. 2019;29:178–188. 10.1016/j.tcb.2018.10.005 30455121

[3] Olgeiser L, Haag C, Boerner S, Ule J, Busch A, Koepke J, et al. The key protein of endosomal mRNP transport Rrm4 binds translational landmark sites of cargo mRNAs. EMBO Rep. 2019;20:e46588. doi: 10.15252/embr.201846588 30552148 PMC6322384

[4] Baumann S, König J, Koepke J, Feldbrügge M. Endosomal transport of septin mRNA and protein indicates local translation on endosomes and is required for correct septin filamentation. EMBO Rep. 2014;15:94–102. doi: 10.1002/embr.201338037 . 24355572 PMC4303453

[5] Müntjes K, Devan SK, Reichert AS, Feldbrügge M. Linking transport and translation of mRNAs with endosomes and mitochondria. EMBO Rep. 2021;22(10):e52445. doi: 10.15252/embr.202152445 . 34402186 PMC8490996

[6] Cioni JM, Lin JQ, Holtermann AV, Koppers M, Jakobs MAH, Azizi A, et al. Late endosomes act as mRNA translation platforms and sustain mitochondria in axons. Cell. 2019;176(1–2):56–72 e15. doi: 10.1016/j.cell.2018.11.030 . 30612743 PMC6333918

[7] Jankowski S, Pohlmann T, Baumann S, Müntjes KM, Devan SK, Zander S, et al. The multi PAM2 protein Upa2 functions as novel core component of endosomal mRNA transport. EMBO Rep. 2019;24:e47381. doi: 10.15252/embr.201847381 31338952 PMC6726905

[8] Devan SK, Schott-Verdugo S, Müntjes K, Bismar L, Reiners J, Hachani E, et al. A MademoiseLLE domain binding platform links the key RNA transporter to endosomes. PLoS Genet. 2022;18(6):e1010269. doi: 10.1371/journal.pgen.1010269 . 35727840 PMC9249222

[9] Hall RA, Wallace EWJ. Post-transcriptional control of fungal cell wall synthesis. Cell Surf. 2022;8:100074. Epub 20220112. doi: 10.1016/j.tcsw.2022.100074 . 35097244 PMC8783092

[10] Sankaranarayanan S, Haag C, Petzsch P, Köhrer K, Matuszynska A, Zarnack K, et al. The mRNA stability factor Khd4 defines a specific mRNA regulon for membrane trafficking in the pathogen *Ustilago maydis*. Proc Natl Acad Sci U S A. 2023;120:e2301731120. doi: 10.1073/pnas.2301731120 . 37590419 PMC10450656

[11] Vollmeister E, Haag C, Zarnack K, Baumann S, König J, Mannhaupt G, et al. Tandem KH domains of Khd4 recognize AUACCC and are essential for regulation of morphology as well as pathogenicity in *Ustilago maydis*. RNA. 2009;15(12):2206–2218. doi: 10.1261/rna.1817609 . 19854870 PMC2779690

[12] Heimel K. Unfolded protein response in filamentous fungi-implications in biotechnology. Appl Microbiol Biotechnol. 2015;99(1):121–32. Epub 20141111. doi: 10.1007/s00253-014-6192-7 . 25384707

[13] Schmitz L, McCotter S, Kretschmer M, Kronstad JW, Heimel K. Transcripts and tumors: regulatory and metabolic programming during biotrophic phytopathogenesis. F1000Res. 2018;7. Epub 20181119. doi: 10.12688/f1000research.16404.1 . 30519451 PMC6248262

[14] Heimel K, Freitag J, Hampel M, Ast J, Bölker M, Kämper J. Crosstalk between the unfolded protein response and pathways that regulate pathogenic development in Ustilago maydis. Plant Cell. 2013;25(10):4262–77. Epub 2013/11/02. doi: 10.1105/tpc.113.115899 . 24179126 PMC3877826

[15] Pinter N, Hach CA, Hampel M, Rekhter D, Zienkiewicz K, Feussner I, et al. Signal peptide peptidase activity connects the unfolded protein response to plant defense suppression by *Ustilago maydis*. PLoS Pathog. 2019;15(4):e1007734. Epub 20190418. doi: 10.1371/journal.ppat.1007734 . 30998787 PMC6490947

[16] Scherer M, Heimel K, Starke V, Kämper J. The Clp1 protein is required for clamp formation and pathogenic development of *Ustilago maydis*. Plant Cell. 2006;18(9):2388–2401. doi: 10.1105/tpc.106.043521 . 16920779 PMC1560919

[17] Heimel K, Scherer M, Schuler D, Kämper J. The *Ustilago maydis* Clp1 protein orchestrates pheromone and b-dependent signaling pathways to coordinate the cell cycle and pathogenic development. Plant Cell. 2010;22(8):2908–22. Epub 20100820. doi: 10.1105/tpc.110.076265 . 20729384 PMC2947178

[18] Hampel M, Jakobi M, Schmitz L, Meyer U, Finkernagel F, Doehlemann G, et al. Unfolded protein response (UPR) regulator Cib1 controls expression of genes encoding secreted virulence factors in *Ustilago maydis*. PLoS ONE. 2016;11(4):e0153861. Epub 20160419. doi: 10.1371/journal.pone.0153861 . 27093436 PMC4836707

[19] Glazier VE, Kaur JN, Brown NT, Rivera AA, Panepinto JC. Puf4 regulates both splicing and decay of HXL1 mRNA encoding the unfolded protein response transcription factor in *Cryptococcus neoformans*. Eukaryot Cell. 2015;14(4):385–95. Epub 20150213. doi: 10.1128/EC.00273-14 . 25681267 PMC4385805

[20] Freitag J, Ast J, Bolker M. Cryptic peroxisomal targeting via alternative splicing and stop codon read-through in fungi. Nature. 2012;485(7399):522–5. Epub 20120523. doi: 10.1038/nature11051 . 22622582

[21] Schueren F, Lingner T, George R, Hofhuis J, Dickel C, Gartner J, et al. Peroxisomal lactate dehydrogenase is generated by translational readthrough in mammals. eLife. 2014;3:e03640. Epub 20140923. doi: 10.7554/eLife.03640 . 25247702 PMC4359377

[22] Bittner E, Stehlik T, Freitag J. Sharing the wealth: The versatility of proteins targeted to peroxisomes and other organelles. Front Cell Dev Biol. 2022;10:934331. Epub 20220926. doi: 10.3389/fcell.2022.934331 . 36225313 PMC9549241

[23] Stiebler AC, Freitag J, Schink KO, Stehlik T, Tillmann BA, Ast J, et al. Ribosomal readthrough at a short UGA stop codon context triggers dual localization of metabolic enzymes in fungi and animals. PLoS Genet. 2014;10(10):e1004685. Epub 20141023. doi: 10.1371/journal.pgen.1004685 . 25340584 PMC4207609

[24] Kremp M, Bittner E, Martorana D, Klingenberger A, Stehlik T, Bölker M, et al. Non-AUG translation initiation generates peroxisomal isoforms of 6-phosphogluconate dehydrogenase in fungi. Front Cell Dev Biol. 2020;8:251. Epub 20200505. doi: 10.3389/fcell.2020.00251 . 32432107 PMC7214817

[25] Cheng AP, Kwon S, Adheshara T, Göhre V, Feldbrügge M, Weiberg A. Extracellular RNAs of plant-colonizing fungi: from molecular mechanisms to crop solutions. Appl Micro Biotech. 2023. doi: 10.1007/s00253-023-12718-7 . 37572124 PMC10485130

[26] Weiberg A, Wang M, Lin FM, Zhao H, Zhang Z, Kaloshian I, et al. Fungal small RNAs suppress plant immunity by hijacking host RNA interference pathways. Science. 2013;342(6154):118–23. Epub 2013/10/05. doi: 10.1126/science.1239705 . 24092744 PMC4096153

[27] He B, Cai Q, Qiao L, Huang CY, Wang S, Miao W, et al. RNA-binding proteins contribute to small RNA loading in plant extracellular vesicles. Nat Plants. 2021;7(3):342–52. Epub 20210225. doi: 10.1038/s41477-021-00863-8 . 33633358 PMC7979528

[28] Kwon S, Rupp O, Brachmann A, Blum CF, Kraege A, Goesmann A, et al. mRNA Inventory of Extracellular Vesicles from *Ustilago maydis*. J Fungi. 2021;7(7). Epub 20210714. doi: 10.3390/jof7070562 . 34356940 PMC8306574

[29] Li G, Dulal N, Gong Z, Wilson RA. Unconventional secretion of *Magnaporthe oryzae* effectors in rice cells is regulated by tRNA modification and codon usage control. Nat Microbiol. 2023. Epub 20230810. doi: 10.1038/s41564-023-01443-6 . 37563288

